# GABAergic and glycinergic inhibitory synaptic transmission in the ventral cochlear nucleus studied in VGAT channelrhodopsin-2 mice

**DOI:** 10.3389/fncir.2014.00084

**Published:** 2014-07-24

**Authors:** Ruili Xie, Paul B. Manis

**Affiliations:** ^1^Department of Otolaryngology/Head and Neck Surgery, University of North Carolina at Chapel HillChapel Hill, NC, USA; ^2^Department of Cell Biology and Physiology, University of North Carolina at Chapel HillChapel Hill, NC, USA

**Keywords:** IPSC, target-specific inhibition, bushy, multipolar, stellate

## Abstract

Both glycine and GABA mediate inhibitory synaptic transmission in the ventral cochlear nucleus (VCN). In mice, the time course of glycinergic inhibition is slow in bushy cells and fast in multipolar (stellate) cells, and is proposed to contribute to the processing of temporal cues in both cell types. Much less is known about GABAergic synaptic transmission in this circuit. Electrical stimulation of the auditory nerve or the tuberculoventral pathway evokes little GABAergic synaptic current in brain slice preparations, and spontaneous GABAergic miniature synaptic currents occur infrequently. To investigate synaptic currents carried by GABA receptors in bushy and multipolar cells, we used transgenic mice in which channelrhodopsin-2 and EYFP is driven by the vesicular GABA transporter (VGAT-ChR2-EYFP) and is expressed in both GABAergic and glycinergic neurons. Light stimulation evoked action potentials in EYFP-expressing presynaptic cells, and evoked inhibitory postsynaptic potentials (IPSPs) in non-expressing bushy and planar multipolar cells. Less than 10% of the IPSP amplitude in bushy cells arose from GABAergic synapses, whereas 40% of the IPSP in multipolar neurons was GABAergic. In voltage clamp, glycinergic IPSCs were significantly slower in bushy neurons than in multipolar neurons, whereas there was little difference in the kinetics of the GABAergic IPSCs between two cell types. During prolonged stimulation, the ratio of steady state vs. peak IPSC amplitude was significantly lower for glycinergic IPSCs. Surprisingly, the reversal potentials of GABAergic IPSCs were negative to those of glycinergic IPSCs in both bushy and multipolar neurons. In the absence of receptor blockers, repetitive light stimulation was only able to effectively evoke IPSCs up to 20 Hz in both bushy and multipolar neurons. We conclude that local GABAergic release within the VCN can differentially influence bushy and multipolar cells.

## Introduction

Inhibition plays multiple roles in sensory information processing that depend on the spatial arrangement of inhibitory circuits relative to the sensory map, and the time course of synaptic currents. Thus, inhibition can shape sensory response areas, as well as define the temporal patterns and rates of ongoing responses. In the auditory brainstem, local and projection circuits utilize both glycine and GABA as transmitters. For example, descending pathways from subnuclei of the superior olivary complex to the cochlear nuclei include both glycincergic and GABAergic components (Ostapoff et al., 1997). Local circuits within the cochlear nuclei can be glycinergic, GABAergic, or utilize both transmitters (Kolston et al., 1992). In the ventral cochlear nucleus (VCN), the synaptically mediated conductances and kinetics of glycine receptors have been extensively studied (Wu and Oertel, 1986; Harty and Manis, 1996; Ferragamo et al., 1998; Harty and Manis, 1998; Xie and Manis, 2013). The glycinergic synaptic conductances of the two principal cell types in the VCN, the bushy and multipolar cells, have very different kinetics (Xie and Manis, 2013), suggesting a critical role for the time course of inhibition in auditory processing by the cochlear nuclei. Whether there are also differences in GABA_A_ synaptic currents between these two principal cell types is not known.

Synaptically-mediated conductances associated exclusively with GABA_A_ receptors have been difficult to detect in the VCN, possibly because such synapses are small and relatively rare compared to glycinergic synapses (Juiz et al., 1996). Electrical stimulation of the auditory nerve or the tuberculoventral pathway from the dorsal cochlear nucleus (DCN) evokes little or no GABAergic synaptic current in VCN neurons in brain slices (Xie and Manis, 2013), and spontaneous GABAergic miniature synaptic currents are observed infrequently when glycinergic receptors are blocked with strychnine. However, in VCN slices, GABA_A_ conductances can be activated pharmacologically (Wu and Oertel, 1986; Milenković et al., 2007), and block of GABA receptors suggests a role in gating polysynaptic activity (Ferragamo et al., 1998). Furthermore, neurotransmitter binding suggests that GABA receptors are present in the VCN (Frostholm and Rotter, 1986; Juiz et al., 1994). Anatomical studies have revealed GAD-positive terminals on the soma and proximal dendrites of most cochlear nucleus neurons (Adams and Mugnaini, 1987; Moore and Moore, 1987; Roberts and Ribak, 1987; Saint Marie et al., 1989). Iontophoresis of GABA and muscimol *in vivo* has clearly demonstrated that GABA receptor activation can inhibit the acoustic responses of VCN neurons (Caspary et al., 1979, 1994; Palombi and Caspary, 1992; Ebert and Ostwald, 1995a,b; Backoff et al., 1999). A common theme is that GABA suppresses spontaneous activity more than evoked activity. GABA antagonists also modify the responses to sinusoidally amplitude-modulated tones and to tones in noise (Backoff et al., 1999; Gai and Carney, 2008), suggesting a functional role for GABA in enhancing information about envelopes, and in spectral processing in circuits of the VCN. The robust and fairly consistent effects seen *in vivo* however stand in contrast to an absence of synaptically-evoked GABA responses in *in vitro* experiments.

There are two potential explanations for the differences between the *in vitro* and *in vivo* evidence for GABA_A_ mediated synaptic inhibition in the VCN. First, *in vivo*, pharmacological agonists and antagonists can activate or inactivate the GABAergic circuits, because all of the incoming pathways are intact and functional, regardless of whether they originate within the cochlear nuclei or from descending projections. In contrast, in brain slices, such circuits may be completely or partially missing because they arise from outside the nucleus, or because the fibers run in different planes than the primary slice orientation. Thus, the receptors would remain functional, but stimulation at an appropriate site to activate specific axons from the extrinsic circuits may be difficult to achieve. Second, there are few GABAergic neurons in the VCN, and few GABAergic neurons from the surrounding granule cell regions or the DCN project into the VCN, so local stimulation of the auditory nerve root region or the DCN is not likely to consistently reveal GABAergic inhibition. In the present study, we have used a mouse line in which channelrhodopsin-2 (ChR2) is expressed in neurons under control of the vesicular GABA transporter (VGAT) promoter. VGAT is expressed in glycinergic, GABAergic, and mixed GABAergic-glycinergic synapses (Dumoulin et al., 1999), and is expressed by both glycinergic and GABAergic neurons in the cochlear nuclei (Wang et al., 2009). As a result, in the VGAT-ChR2 mice, optical stimulation can be used to selectively stimulate both local neurons, as well as axons of VGAT-ChR2 expressing distantly-located cells that may project into the nuclei. Using this approach, we have characterized and compared the GABAergic and glycinergic synaptic potentials and conductances in VCN bushy and planar multipolar cells.

## Materials and methods

**Figure 1:**
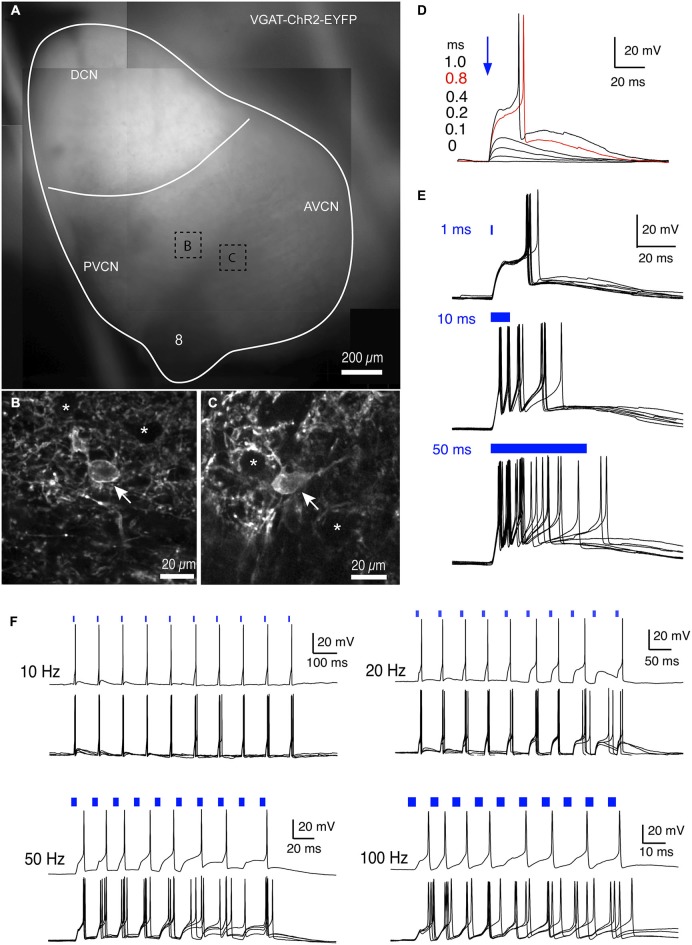
**Photostimulation drives excitatory responses in EYFP expressing cells in VGAT-ChR2-EYFP mice. (A)** Expression pattern of ChR2 in cochlear nucleus as visualized by EYFP fluorescence. Notice that expression is absent in the 8th nerve root region, moderate in anteroventral cochlear nucleus (AVCN) and posteroventral cochlear nucleus (PVCN), and high in the DCN. The image is a mosaic assembled from different areas of the cochlear nuclei. **(B–C)** Multiphoton images of EYFP expressing cells from areas as marked in **(A)**. Expression of the EYFP-ChR2 construct is present in both membrane and cytoplasm. Arrows mark expressing neurons. Asterisks mark non-expressing cells whose soma is surrounded by expressing terminals. **(D)** Example responses from an EYFP-ChR2 expressing cell to different durations of 470 nm illumination from 0 (no light) to 1.0 ms. The threshold of the light duration was 0.8 ms in this cell, which evoked an action potential as shown in red. All sub-threshold traces are averages of 5–10 trials; traces with spikes are single trials. **(E)** Longer duration illumination reliably evoked a single spike or trains of spikes (same cell in **(D)**). Each plot shows the responses to 5–10 trials. **(F)** Ten 2-ms light pulses at 10, 20, 50, and 100 Hz evoke trains of spikes. Top: single trial; bottom: superimposed traces from four trials. Note that tonic firing is evoked at higher frequencies, although the cell no longer entrains to individual flashes.

VGAT-ChR2-EYFP mice (B6.Cg-Tg(Slc32a1-COP4*H134R/EYFP)8Gfng/J; (Zhao et al., 2011)) were purchased from Jackson Laboratories (stock #014548) and maintained in our breeding colony. The mice incorporate a BAC transgene that expresses ChR2 and enhanced yellow fluorescent protein (EYFP) under the control of the VGAT promoter. Because ChR2 is fused to EYFP, EYFP fluorescence directly reports the cellular localization of ChR2 (see Figure 1). All animal procedures were approved by the University of North Carolina Internal Animal Concerns and Use Committee (IACUC).

Slice preparation follows the approach used in our recent studies (Wang and Manis, 2005; Xie and Manis, 2013). Mice were anesthetized with an intraperitoneal injection of 100 mg/kg ketamine and 10 mg/kg xylazine, decapitated, and the brain dissected and placed in a warmed (34°C) artificial cereberospinal fluid (ACSF) solution. The ACSF contained (in mM): 122 NaCl, 3 KCl, 1.25 NaH_2_PO_4_, 25 NaHCO_3_, 20 glucose, 3 myo-inositol, 2 sodium pyruvate, 0.4 ascorbic acid, 2.5 CaCl_2_, and 1.5 MgSO_4_, saturated with 95% O_2_-5% CO_2_. After taking a thin slice that removes the external granule cell layer over the anterior VCN, a single 350 µm thick parasagittal slice of the cochlear nuclei that includes the VCN and DCN was cut and incubated in ACSF at 34°C for about 1 h before recordings commenced. During recording, slices were placed in a fast-flow chamber (Warner Instruments) on a fixed stage (34°C), and visualized under both brightfield and fluorescence optics (Zeiss FS2 microscope). Fluorescence illumination to detect cells expressing EYFP was provided by a 505 nm LED (Phillips).

Some experiments were performed on a separate recording system that permitted 2-photon illumination as well as widefield fluorescence. For the overall evaluation of EYFP expression, standard fluorescence (1-photon) was imaged using illumination from a 530 nm LED (Phillips Luxeon) through a standard Zeiss filter set. Images were captured with a Photometrics EM512 CCD camera. For 2-photon illumination, a custom system built around a Ti-Sapphire laser (Coherent Chameleon Ultra II) was coupled through a Pockels cell (Conoptics) and scan mirrors (Cambridge 6210 H) into a modified epi-illumination train on a Zeiss FS2 microscope through a dichroic mirror (FF670-SDi01, Semrock) and a 630 × 0.90 nA water immersion objective (Zeiss). The collected fluorescence was passed through a short-pass filter (FF01-680/SP-25, Semrock), followed by a narrowband filter (FF03-525/50–25 or FF02-617/73–25; Semrock) depending on the fluorophore to be detected. The fluorescence was detected by a cooled Ga-As photomultiplier (Hamamatsu H7422P50) and amplified with a custom wide-band current-to-voltage converter before being digitized.

Photostimulation in these experiments was provided by gating the light from a 470 nm LED coupled through the epi-illumination ports of the microscopes. The light from the LED was passed through a lens and a pair of dichroic mirrors. The lens was adjusted so that the illumination was visually uniform at the specimen plane. Photostimulation took place through a 40 × 0.75 nA objective, focused on the cell of interest. To measure the illuminated area, we soaked a strip of nitrocellulose filter paper (Schleicher and Schuell) with a ~1% solution of Lucifer Yellow Cadeverine Biotin X (Life Technologies) in water, then dried the paper, and sandwiched it between two coverslips. The illumination from the objective was used to bleach the dye over about 5 min, after which a low-magnification image of the bleached spot was digitized, and the diameter of the bleached area measured. The diameter of the circular area illuminated with the 40X objective was 780 µm at the focal plane. Because cells were recorded from the center of the visible field, the illumination was roughly centered over the cell. The incident light at the specimen plane was ~0.8 mW (Newport 1917-R power meter with 818UV/DB detector), corresponding to an irradiance of ~1.7 mW/mm^2^. For a few experiments we used a 63X objective to record the photostimulation evoked response patterns in ChR2-expressing neurons (Figure 1). For this objective, the diameter of the illuminated area at the focal plane was measured to be 280 µm, and the total incident power was 0.110 mW, corresponding to an irradiance of ~2.1 mW/mm^2^.

Whole-cell tight seal recordings were made with Multiclamp 700A and B amplifiers, using KG-33 glass (King Glass, Claremont, CA) or 1.2 mm glass (Sutter). Pipettes were backfilled with one of three electrode solutions, and had open tip resistances of 4–7 MOhms. The K-gluconate based electrode solution used for current clamp recordings contained (in mM): 126 K-gluconate, 6 KCl, 2 NaCl, 10 HEPES, 0.2 EGTA, 4 Mg-ATP, 0.3 Tris-GTP, and 10 Tris-phosphocreatine, with pH adjusted to 7.2 with KOH. Two different Cs-based electrode solutions were used for voltage clamp recordings. One had a low chloride concentration (8 mM; calculated E_Cl_ = −71.1 mV), and contained (in mM): 130 CsMetSO_3_, 5 CsCl, 5 EGTA, 10 HEPES, 4 MgATP, 0.3 Tris-GTP, 10 Tris-phosphocreatine, and 3 QX-314 (chloride salt), with pH adjusted to 7.2 with CsOH. The second Cs-based electrode solution had high chloride concentration (38 mM; calculated E_Cl_ = −31.1 mV) and contained (in mM): 105 CsMetSO_3_, 35 CsCl, 5 EGTA, 10 HEPES, 4 MgATP, 0.3 Tris-GTP, 10 Tris-phosphocreatine, and 3 QX-314 (chloride salt), with pH adjusted to 7.2 with CsOH. For voltage clamp recordings, compensation of >75% was applied on-line. Junction potentials are calculated to be −12 mV for the K-gluconate based electrode solution, −8 mV for the Cs-based electrode solution with 8 mM chloride and −7 mV for the Cs-based electrode solution with 38 mM chloride. All reported voltages have been corrected for the appropriate junction potentials. Cells were characterized in current clamp by their firing patterns, and morphologically by their patterns of dendritic branching when filled with Lucifer Yellow or AlexaFluor (594, 488). Cells recorded in voltage clamp were identified by their dendritic branching patterns in conjunction with the time course of sIPSCs (Xie and Manis, 2013). Cells with 1–2 short, stout dendrites and a profusion of fine dendrites at the end of each primary dendrite were classified as bushy cells. Cells which had 2–5 long primary dendrites that were oriented parallel to the fascicles of the auditory nerve fibers were classified as planar multipolar (T-stellate) cells. Cells that had 2–5 long primary dendrites, at least some of which crossed the fascicles of auditory nerve fibers at an oblique angle were classified as radiate multipolar cells.

### Analysis

IPSC decay time constants were calculated by fitting the decay phase of IPSCs with single or double exponential functions. Double exponential fits were used only when the χ^2^ value from single exponential fits. Weighted decay time constants (*τ_w_*) were calculated from double exponential fits as previous described (Xie and Manis, 2013) using the following function: *τ_w_* = A_1_ * *τ*_1_ + A_2_ * *τ*_2_, where A_1_ and A_2_ are the normalized amplitude of each component and A_1_ + A_2_ = 1.

Reversal potentials were measured using Cs^+^ electrodes containing 38 mM Cl^−^. For these measurements, cells were held in voltage-clamp at −57 mV, and stepped from −107 to +13 mV (corrected for a −7 mV junction potential) in 10 mV steps for 750–850 ms. A 20 ms maximal light flash was delivered 600 ms after the onset of the voltage step. The presentation of voltages was randomized, and the entire sequence was repeated four times, with a 10 s interval between trials. Reversal potentials were measured in control solution, following exposure to 2 µM strychnine, and following the addition of both 10 µM SR95531 and 2 µM strychnine. No evoked currents were seen with the combination of strychnine and SR95531, except in one radiate multipolar cell, where light-evoked ChR2 currents reversing at +4 mV were observed (data from this cell is not included in the multipolar population analyzed in the Results section). Not all voltage-gated currents were blocked with the Cs^+^ electrode solution, so we calculated the contribution of the voltage-gated current to the overall response. To accomplish this, the time course of voltage-gated current for each trace, beginning 200 ms before the light flash, and ending 150–250 ms after the flash onset, excluding a 100 ms window starting at the time of the flash, was fit to a cubic polynomial. The estimated current during the flash was then interpolated from the polynomial fit, and subtracted from the evoked response. The evoked response was calculated as the mean current over 16 ms beginning 4 ms after the flash onset. The command voltage was corrected for the uncompensated portion of the series resistance (compensation of 75% was used, and the uncompensated series resistance ranged from 1.3 to 2.5 MΩ) and the total (unsubtracted) current. The resulting current-voltage relationship, which often exhibited a modest outward rectification, was then fit to a cubic spline function. The reversal potential was calculated from the zero current intercept, and the synaptic conductance was calculated from the slope at −60 mV.

### Reagents

Strychnine (2 µM, Sigma-Aldrich) was bath applied to block glycine receptors. SR95531 (10 µM, Sigma-Aldrich) was bath applied to block GABA_A_ receptors. CNQX (5 µM, Tocris Bioscience) was bath applied to block AMPA receptors. Tetrodotoxin (1 µM, Sigma-Aldrich) was used to block voltage-gated sodium channels. All salts used to make the ACSF were purchased from Sigma-Aldrich.

### Software and statistical analysis

All recordings, control of optical stimulation and both CCD and laser imaging, were made using custom software, Acq4 (Campagnola et al., 2014). Data were analyzed using Igor Pro (version 6.3.4.0, WaveMetrics), and custom routines in Acq4 using the Python libraries numpy (version 1.8.0)^1^ and scipy (version 0.13.3).^2^ Statistical analyses were performed using GraphPad Prism (GraphPad Software Version 5.01 and 6.0, San Diego, CA). Group results were compared using unpaired or paired student’s *t*-tests, or using a two-way repeated measures ANOVA. Data are presented as mean ± standard deviation.

## Results

### Photostimulation generates depolarization and spikes in eYFP expressing cells in VGAT-ChR2-eYFP mice

We examined the expression pattern of ChR2-eYFP in the cochlear nucleus of the VGAT-ChR2-EYFP mice. As ChR2 is expressed in conjunction with EYFP, the expression can be visualized under 505–530 nm light that excites EYFP (Figures 1A–C). As shown in Figure 1A, the expression was high in the DCN, moderate in the anteroventral cochlear nucleus (AVCN) and posteroventral cochlear nucleus (PVCN), and very low in the auditory nerve root area. This pattern is consistent with the distribution of inhibitory neurons in the cochlear nuclei, in which DCN contains the most inhibitory neurons including cartwheel and tuberculoventral neurons, AVCN and PVCN only contain scattered inhibitory (radiate multipolar, or D-stellate) neurons, and the auditory nerve area is made up of excitatory nerve fibers with few inhibitory neurons. Multiphoton imaging of individual neurons expressing EYFP in the AVCN and PVCN (Figures 1B,C, arrows) revealed the ChR2 construct in both the cell membrane and cytoplasm. The majority of the neurons in the AVCN and PVCN, however, do not express ChR2 as shown by the dark cells (marked with asterisks) in Figures 1B,C. These non-expressing cells are likely excitatory neurons including bushy and planar multipolar (T-stellate) neurons. Interestingly, the soma of these neurons is often surrounded by a ring of fluorescent terminals, suggesting that these cells receive synaptic inputs from expressing inhibitory neurons.

We next studied how expressing neurons in the AVCN respond to photostimulation using light pulses at 470 nm with different durations (Figures 1D,E), delivered through a 63X objective focused on the recorded cell. Current clamp recordings were obtained using standard K-gluconate electrode solution. Light pulses with different durations (Figure 1D) evoked depolarization and action potentials in the expressing neurons. The size of the depolarization increased with increasing light duration until it reached action potential threshold. The threshold duration of light ranged from 0.4 to 1.0 ms with an average of 0.8 ± 0.3 ms (*n* = 4). Suprathreshold light pulses reliably drove spikes in expressing neurons, and prolonged light pulses (10 and 50 ms in Figure 1E) generated multiple spikes. Expressing neurons were also able to fire trains of spikes in response to trains of brief light pulses at 10–100 Hz (Figure 1F), although entrainment was only seen for the first few pulses at 50 and 100 Hz, after which firing continued at a lower rate than the pulse rate. In contrast, non-expressing neurons always responded to light pulses with IPSPs and never responded with EPSPs, depolarization or action potentials. These results suggests that inhibitory neurons expressing VGAT can be selectively stimulated, and further that non-expressing cells are excitatory neurons that receive inhibitory input from the expressing cells.

### The strength of GABAergic relative to glycinergic inhibition is larger in multipolar than bushy cells

**Figure 2:**
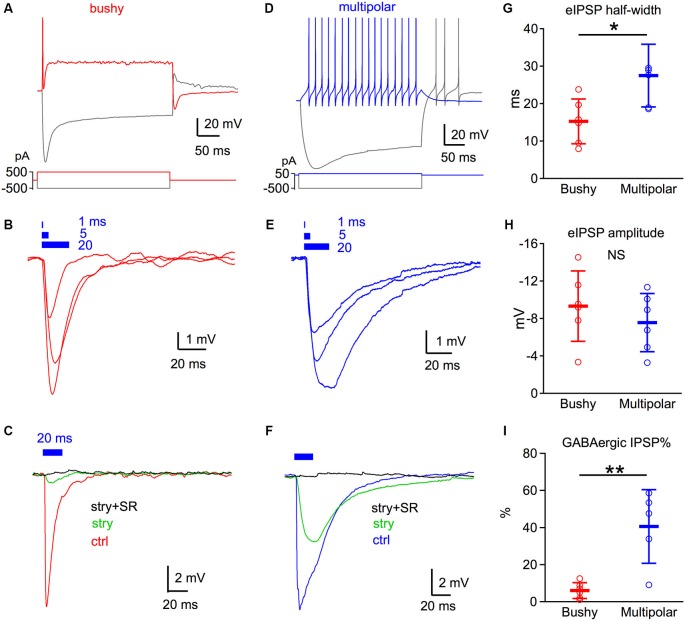
**GABAergic inhibition is weak in bushy but strong in multipolar neurons**. **(A)** Discharge pattern of a bushy neuron to direct current injection. The bushy neuron fires only one or a few spikes with depolarizing current injections. **(B)** Light pulses at different durations evoke brief IPSP responses in bushy neurons. Each trace is an average of six trials. Blue bars on top mark the timing of the light pulses with duration of 1, 5 and 20 ms. **(C)** Strychnine (stry) blocks the majority of the IPSP evoked by 20 ms light pulses in bushy neurons. Addition of SR95531 (stry+SR) fully blocks light evoked IPSPs. Traces are averages of 10 trials. Data in **(A–C)** are from the same bushy neuron. **(D)** Discharge pattern of an example multipolar neuron to direct current injections. Multipolar neurons fire a regular train of spikes throughout the current injection. **(E)** Light pulses at durations of 1, 5 and 20 ms evoke IPSPs in multipolar neurons. Note that the IPSPs have a wider half-width than those of bushy neurons in **(B)**. **(F)** Strychnine only blocks about half of light evoked IPSP. The remainder of the current is fully blocked by the further addition of SR95531. Data in **(D–F)** are from the same multipolar neuron. **(G–I)** Summary data of the eIPSP half-width **(G)**, eIPSP amplitude **(H)** and percentage of GABAergic IPSP **(I)**. * *p* < 0.05; ** p < 0.01. Data is plotted as mean ± S.D.

We next characterized the light evoked inhibitory responses using current clamp recordings from non-expressing neurons in AVCN. All non-expressing neurons were classified into two cell types based on their characteristic firing patterns to depolarizing current injections. Bushy neurons fire only one or a few transient spikes after the onset of the depolarizing current injection (Figure 2A), while multipolar (stellate) neurons fire tonically throughout the duration of the current injection (Figure 2D). The multipolar neurons are primarily planar multipolar (T-stellate) neurons, because these are excitatory neurons that do not express ChR2 in this mouse.

Brief 470 nm light pulses evoked IPSPs in both bushy and multipolar neurons (Figures 2B,E). In bushy neurons, light pulses of different durations evoked IPSPs that decayed very rapidly (Figure 2B). In contrast, light evoked IPSPs in the multipolar cells were longer lasting (Figure 2E). The average half-width of the IPSPs evoked by 20 ms light pulse was 15.25 ± 6.0 ms (*n* = 6) in bushy cells, but was 27.5 ± 8.4 ms (*n* = 6) in multipolar neurons (Figure 2G; unpaired *t*-test: *t*_10_ = 2.92, *p* = 0.015). The shorter IPSP half-width in bushy neurons is likely due to their faster membrane time constant compared to multipolar neurons (Manis and Marx, 1991; Francis and Manis, 2000; Xie and Manis, 2013).

We then isolated the glycinergic and GABAergic components of the light evoked IPSPs using strychnine and SR95531. Under control condition, 20 ms light pulses evoked IPSPs with similar amplitude in both bushy (−9.3 ± 3.8 mV, *n* = 6) and multipolar neurons (−7.6 ± 3.1 mV, *n* = 6) (Figure 2H; unpaired *t*-test: *t*_10_ = 0.89, *p* = 0.393). Bath application of 2 µM strychnine reduced IPSP amplitudes by 93.9 ± 4.3% (*n* = 6) in bushy neurons, but only by 59.5 ± 19.9% (*n* = 5) in multipolar neurons (Figures 2C,F; unpaired *t*-test: *t*_9_ = 4.18, *p* = 0.0024). The remaining IPSPs in both cell types were fully blocked with a subsequent application of 10 µM SR95531 in the presence of strychnine. Previously, only glycinergic IPSPs have been seen (Wu and Oertel, 1986; Xie and Manis, 2013) following electrical stimulation. Thus, our new results demonstrate the presence of functional synaptically-evoked GABAergic IPSPs in VCN neurons in slices.

### The time course of GABAergic inhibition is similar in bushy and multipolar neurons

**Figure 3:**
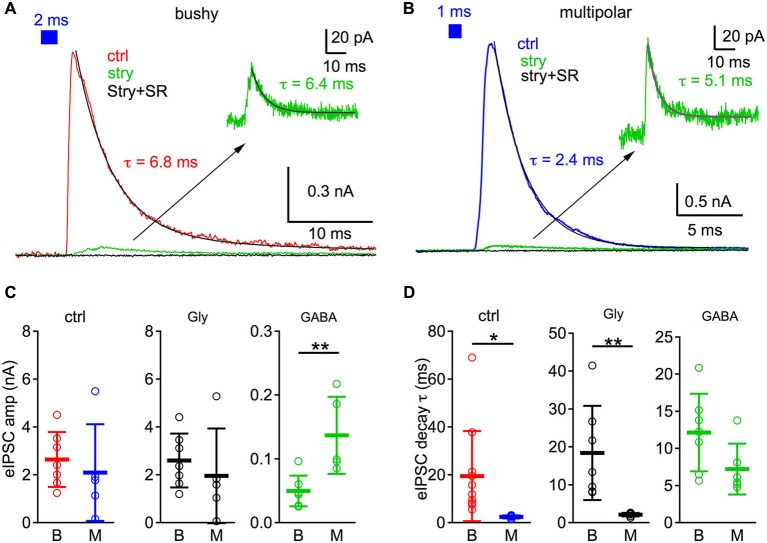
**Kinetics of GABAergic and glycinergic IPSCs in bushy and multipolar neurons**. **(A)** A 2 ms light pulse (blue bar on top) evokes IPSCs in a bushy neuron. The IPSC is mostly blocked by strychnine (stry) and is fully blocked by a combination of strychnine and SR95531 (Stry + SR). Inset: magnified IPSC trace after strychnine block. The weighted decay time constants of the IPSCs are obtained by fitting the IPSC decay with double exponential functions (black curves) under both control and stry conditions. **(B)** 1 ms light pulse evokes IPSCs in a multipolar neuron. Plots are organized the same as in **(A)**. Decay time constants of IPSCs are obtained by fitting the IPSC decay with single exponential functions (black curves). Traces in both **(A)** and **(B)** are averages of 10 trials. **(C)** Comparison of the light evoked IPSC amplitudes between bushy and multipolar neurons including the control IPSC amplitude (ctrl), glycinergic IPSC component (Gly), and GABAergic IPSC component (GABA). Abcissa: B: bushy neurons; M: multipolar neurons. **(D)** Comparison of the eIPSC decay time constants. * *p* < 0.05; ** *p* < 0.01. Data is plotted as mean ± S.D.

In a separate population of cells, we investigated the kinetics of the light evoked synaptic currents under voltage clamp (Figure 3). Recordings were made using Cs-based electrode solution (8 mM Cl^−^) with 3 mM QX-314 to block potassium and sodium channels and improve clamp quality. Cells were held at **+**42 mV so that the IPSCs were large and outward. Light pulses of 1 or 2 ms were used to evoke repeatable single spikes in presynaptic inputs (Figures 1D,E), to help minimize the possibility that the kinetics of evoked IPSCs were contaminated by multiple synaptic events.

**Figure 4:**
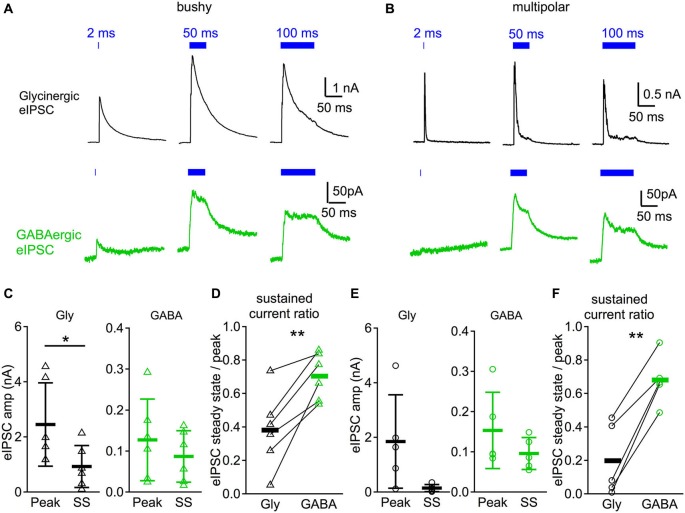
**Glycinergic IPSCs are phasic compared to GABAergic IPSCs in response to sustained illumination. (A)** IPSC responses evoked by different durations of light (2, 50, and 100 ms) in a bushy neuron. Notice that the glycinergic IPSCs peak shortly after the onset of the light pulse and decay rapidly even though the illumination is still on, whereas GABAergic IPSCs show little decay in amplitude. **(B)** Light evoked IPSC responses in a multipolar neuron. Traces in both **(A)** and **(B)** are averages of 10 trials. **(C)** Summary of the IPSC peak amplitudes (peak) and steady state amplitudes (SS) in bushy neurons. In **(C-F)**: Gly: glycinergic IPSCs; GABA: GABAergic IPSCs. **(D)** Summary of the sustained current ratio in bushy neurons. **(E)** Summary of the IPSC peak amplitudes and steady state amplitudes in multipolar neurons. **(F)** Summary of the sustained current ratio in multipolar neurons. In **(D)** and **(F)**: each connected pair represents a single neuron; black bar marks the average of the group. * *p* < 0.05; ** *p* < 0.01.

The amplitudes of light evoked IPSCs in bushy and multipolar neurons were not different under control conditions, similar to the results for IPSPs. The peak IPSC amplitude was 2.65 ± 1.15 nA (*n* = 7) in bushy neurons and 2.10 ± 2.02 nA (*n* = 5) in multipolar neurons (Figure 3C; unpaired *t*-test: *t*_10_ = 0.60, *p* = 0.56). As shown in Figures 3A,B, strychnine blocked most of the IPSC in both bushy and multipolar neurons. We measured the amplitude of the GABAergic IPSCs (in the presence of strychnine) and of the glycinergic IPSCs (computed as the difference between control IPSCs and strychnine-resistant IPSCs). There was no significant difference in the glycinergic IPSC amplitudes (bushy: 2.60 ± 1.13 nA, *n* = 7; multipolar: 1.96 ± 1.98 nA, *n* = 5; Figure 3C; unpaired *t*-test: *t*_10_ = 0.71, *p* = 0.49) between two cell types. However, the GABAergic IPSCs were significantly smaller in bushy neurons (50 ± 24 pA, *n* = 7) than in multipolar neurons (137 ± 61 pA, *n* = 5) (Figure 3C; unpaired *t*-test: *t*_10_ = 3.50, *p* = 0.0057), consistent with the IPSP data (Figure 2I). The small percentage of GABAergic IPSC components measured here with short light pulses (compare to IPSP components in Figure 2I) suggests that GABAergic inhibition is more effectively activated with long light stimulation, which is also demonstrated in Figure 4.

We next measured the decay phase of IPSCs by fitting with double exponential functions in bushy neurons (Figure 3A) and single exponential functions in multipolar neurons (Figure 3B). Weighted decay time constants were then calculated in bushy cells for comparison. As shown in Figure 3D, light evoked IPSCs under control conditions were significantly slower in bushy neurons (weighted decay time constant: 19.5 ± 18.9 ms, *n* = 11) than multipolar neurons (decay time constant: 2.4 ± 0.7 ms, *n* = 6) (unpaired *t*-test: *t*_15_ = 2.19, *p* = 0.045). This difference persisted when pharmacologically isolated glycinergic IPSCs were measured (bushy glycinergic IPSC decay: 18.4 ± 12.5 ms, *n* = 7; multipolar glycinergic IPSC decay: 2.1 ± 0.5, *n* = 6; unpaired *t*-test: *t*_11_ = 3.19, *p* = 0.0087). The fast kinetics of glycinergic IPSCs in multipolar cells is consistent with our previous observations with electrically evoked IPSCs and spontaneous mIPSCs (Xie and Manis, 2013). In the presence of strychnine, the isolated GABAergic component showed a trend for bushy cells (12.2 ± 5.2 ms, *n* = 7) to have a slower decay time constant than the multipolar neurons (7.2 ± 3.4, *n* = 6), however, the difference was not statistically significant (Figure 3D; unpaired *t*-test: *t*_11_ = 1.97, *p* = 0.074). The results suggest that GABAergic inhibition in bushy and multipolar neurons, unlike glycinergic inhibition (Xie and Manis, 2013), does not have widely different kinetics in bushy and multipolar cells.

### Glycinergic IPSCs are phasic whereas GABAergic IPSCs have a large tonic component in response to sustained illumination

We further studied the IPSC kinetics in response to longer duration illumination. Glycinergic and GABAergic IPSCs were pharmacologically isolated with strychnine and SR95531 as above. As shown in Figures 4A,B, both glycinergic IPSCs and GABAergic IPSCs peaked in amplitude shortly after the onset of the light stimulation. The amplitude of glycinergic IPSCs, however, decayed rapidly, whereas GABAergic IPSCs showed less decay and exhibited sustained currents until the end of the light stimulation.

To quantify the magnitude of the current decrease (which we term “sustained current ratio”), we calculated the peak and steady state IPSC amplitudes in response to 50 ms light pulses. The steady state IPSC amplitude was measured from the average current during the last 10 ms of the stimulation. The sustained current ratio of the IPSCs was then calculated as the steady state divided by the peak IPSC amplitude. As shown in Figure 4C, glycinergic IPSCs in bushy neurons showed significantly larger peak amplitudes (2.45 ± 1.51 nA, *n* = 6) than steady state IPSC amplitudes (0.93 ± 0.76 nA, *n* = 6) (paired *t*-test: *t*_5_ = 3.95, *p* = 0.011). In contrast, there was no significant difference in the GABAergic IPSCs between the peak (127 ± 99 pA, *n* = 6) and steady state amplitudes (87 ± 63 pA, *n* = 6) (paired *t*-test: *t*_5_ = 2.12, *p* = 0.088). The computed sustained current ratio was significantly smaller for glycinergic IPSCs (0.38 ± 0.23, *n* = 6) than for GABAergic IPSCs (0.70 ± 0.14, *n* = 6) (Figure 4D; paired *t*-test: *t*_5_ = 4.51, *p* = 0.0063). In multipolar neurons, the glycinergic IPSC had a peak amplitude of 1.85 ± 1.71 nA (*n* = 5) and a steady state amplitude of 0.15 ± 0.13 nA (*n* = 5) (Figure 4E; paired *t*-test: *t*_4_ = 2.22, *p* = 0.090). The GABAergic IPSC had peak amplitude of 153 ± 95 pA (*n* = 5) and steady state amplitude of 96 ± 40 pA (*n* = 5) (Figure 4E; Wilcoxon matched pairs test: *p* = 0.063). Although the steady state and peak amplitudes were not significantly different for either glycinergic or GABAergic IPSC components of the multipolar neurons, there was a significant difference in the sustained current ratio between the two (Figure 4F; glycinergic IPSC: 0.20 ± 0.22; GABAergic IPSC: 0.68 ± 0.15, *n* = 5; paired *t*-test: *t*_4_ = 7.71, *p* = 0.0015). Therefore, in both bushy and multipolar neurons, glycinergic IPSCs decrease more over time than GABAergic IPSCs in response to sustained illumination.

### GABAergic and glycinergic IPSCs have different reversal potentials in multipolar neurons

It is known that both GABAergic and glycinergic IPSCs are associated with an increased permeability for chloride ions across the membrane (Eccles et al., 1977; Wu and Oertel, 1986; Bormann et al., 1987; Harty and Manis, 1996). Therefore, we would expect that both types of IPSCs should have the same reversal potentials, close to the equilibrium potential for chloride. In this study, however, we surprisingly found that the glycinergic IPSCs and GABAergic IPSCs possess different reversal potentials.

**Figure 5:**
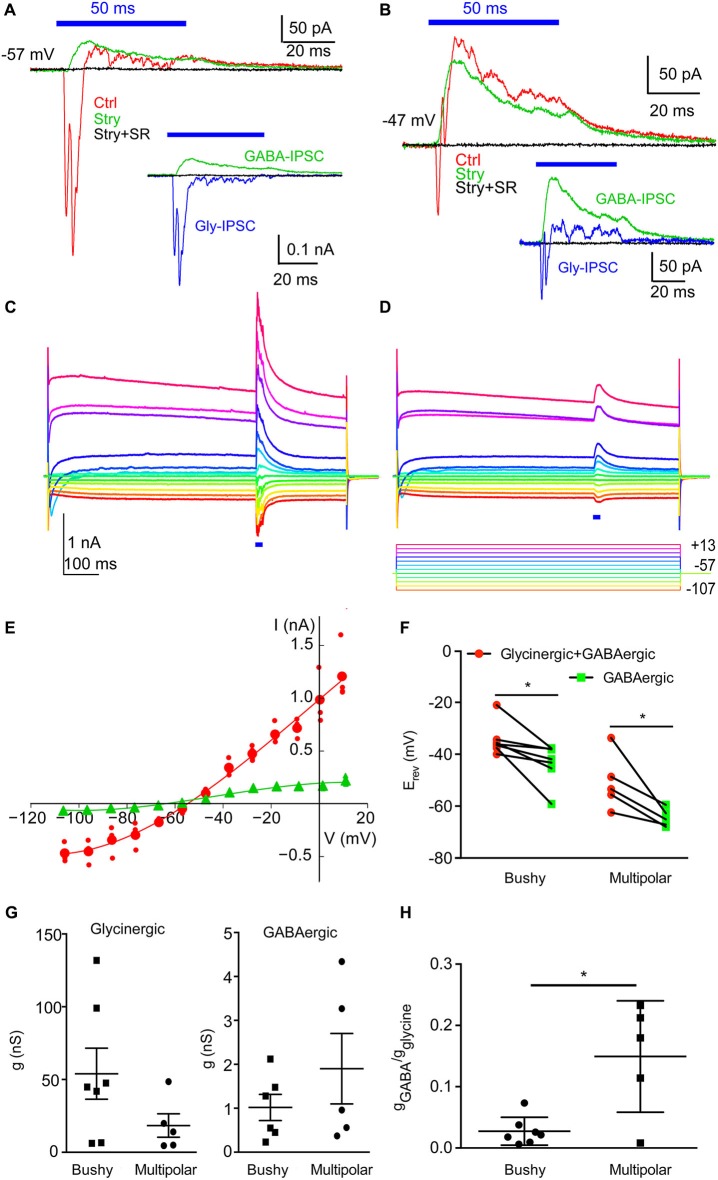
**GABAergic and glycinergic IPSCs show different reversal potentials in multipolar neurons**. **(A)** Multipolar neuron held at −57 mV using 38 mM Cl^−^ electrode solution. IPSC traces show currents in control solution (red), in strychnine (stry, green), and in both strychnine and SR95531 (stry + SR, black). Inset: Isolated glycinergic IPSC (blue), GABAergic IPSC (green), and complete block in stry + SR (black). The glycinergic IPSC is inward, whereas the GABAergic IPSC is outward, suggesting different IPSC reversal potentials. **(B)** IPSCs recorded at holding potential of −47 mV from the same neuron as in **(A)**. Traces in **(A)** and **(B)** are the averages of 20 trials. **(C)** Response to a 20 ms light flash (blue bar below traces) in a voltage-clamped multipolar cell with 38 mM [Cl^−^]_i_ at different voltage steps. The light-evoked currents are superimposed on unblocked currents. Each trace is the average of four trials; peaks of capacitative transients at onset and offset of voltage steps have been clipped. **(D)** Same cell as in **(C)**, in the presence of 2 µM strychnine to isolate the GABAergic component. Voltage steps are indicated below the traces. Current and voltage scales are the same in **(C)** and **(D)**. **(E)** Current-voltage relationship of the light-evoked current (see Section Materials and Methods for analysis details). Large red circles: mean of currents across four trials in control conditions; small circles show responses for individual trials. Red line: cubic spline fit to the data. Large green triangles: responses in the presence of strychnine; small triangles show responses for individual trials. Green line: cubic spline fit to the data. **(F)** Reversal potentials measured as in **(E)** for, for total (glycinergic + GABAergic) currents, and isolated GABAergic currents. Measurements made sequentially in the same cell are connected. Asterisk indicates ANOVA post tests, *p* < 0.05. **(G)** Conductance at −60 mV in individual cells. **(H)** Ratio of GABAergic to glycinergic conductance at −60 mV for individual cells (asterisk, *p* < 0.05).

The difference in the reversal potential between glycinergic and GABAergic IPSCs was initially observed as different directions of currents in three different multipolar neurons when recorded using Cs-based electrode solution containing 38 mM Cl^−^ (Figure 5). At a holding potential of −57 mV, the IPSCs of one multipolar neuron (Figure 5A) under control conditions showed an initial inward current followed by mixed inward and outward currents. When the glycinergic IPSCs were blocked with strychnine, an outward GABAergic IPSC was revealed. This IPSC in turn was completely blocked by the subsequent addition of SR95531, confirming that it was mediated by GABA_A_ receptors. The isolated glycinergic IPSC was entirely inward at this holding potential. Similar features were also apparent in this cell when held at −47 mV (Figure 5B). These results suggest that the reversal potentials of the glycinergic and GABAergic IPSCs are different.

To further clarify the differences in reversal potentials, we made systematic measurements of light-evoked currents at different membrane potentials under control conditions, and in the presence of strychnine alone, and with strychnine and SR95531. Recordings were made using Cs-based electrode solution (38 mM Cl^−^), from 5 multipolar cells and 7 bushy cells in a separate series of experiments. An example of the responses to the voltage steps in the control solution, and the superimposed light evoked response is shown in Figure 5C for one of the multipolar cells. Even with Cs^+^ in the pipette, modest outward currents were evoked by depolarizing voltage steps, and small inward I_h_ currents were observed with hyperpolarizing voltage steps. The voltage-gated currents were subtracted from the light-evoked current as described in the Section Materials and Methods, to measure the isolated current-voltage relationship of the synaptic conductance shown in 5E (red circles). For this cell, the control reversal potential, estimated by interpolation, was −55.6 mV. After the addition of strychnine, the currents were smaller (Figure 5D), and the reversal potential shifted negative to −68.0 mV (Figure 5E, green triangles). As above, the strychnine-insensitive current was blocked by 10 µM SR95531. A summary of the reversal potentials across all cells tested is shown in Figure 5F. A two-way repeated measures ANOVA revealed no interaction between cell types (*F*_1,10_ = 1.011, *p* = 0.34), consistent with the observation that the reversal for GABAergic IPSCs was always negative to that of glycinergic IPSCs in both cell types. The comparison between cell types revealed a significant difference (*F*_1,10_ = 27.48, *p* = 0.0004), as did the comparison between the reversals of GABAergic and glycinergic IPSCs (*F*_1,10_ = 22.05, *p* = 0.0008). Sidak’s multiple comparison corrected post-tests revealed that the reversal potentials for both glycinergic and GABAergic IPSCs were significantly different in bushy cells (*t*_10_ = 2.859, *p* = 0.034; mean difference −8.8 mV, standard error = 3.1) and in multipolar cells (*t*_10_ = 3.732, *p* = 0.0078; mean difference −13.7 mV, standard error = 3.67).

The glycinergic conductance, measured as the slope of the current-voltage relationship at −60 mV, could be quite large in bushy cells (Figure 5G), but this difference was not significantly different than the conductance in multipolar cells (unpaired *t*-test, *t*_10_ = 1.395, *p* = 0.19). The GABAergic conductance similarly was not significantly different between the two cell types (unpaired *t*-test, assuming unequal variances, *t*_4.81_ = 1.087, *p* = 0.33). Figure 5H compares the ratios of the GABAergic to glycinergic conductance in individual cells, and reveals a significant difference between cell types (unpaired *t*-test assuming unequal variances, multipolar ratio 0.15 ± 0.09, bushy ratio 0.027 ± 0.023, *t*_4.36_ = 2.936, *p* = 0.038). This difference is consistent with the larger maximal GABA currents measured in multipolar cells shown in Figure 3. From these observations, we conclude that GABAergic IPSCs reverse at a potential negative to glycinergic IPSCs in both bushy and multipolar cells. These results also show that that the reversal for glycinergic IPSCs in bushy cells is not different than that expected from the equilibrium potential for Cl^−^ (one-sample *t*-test, −35.4 ± 6.8 vs. −31.1 mV, *t*_6_ = 1.668, *p* = 0.15), whereas that for glycinergic IPSCs in multipolar cells is significantly negative to the expected reversal potential (one-sample *t*-test, −50.8 ± 10.7 vs. −31.1 mV, *t*_4_ = 4.098, *p* = 0.015).

### GABAergic IPSCs are blocked by tetrodotoxin

**Figure 6:**
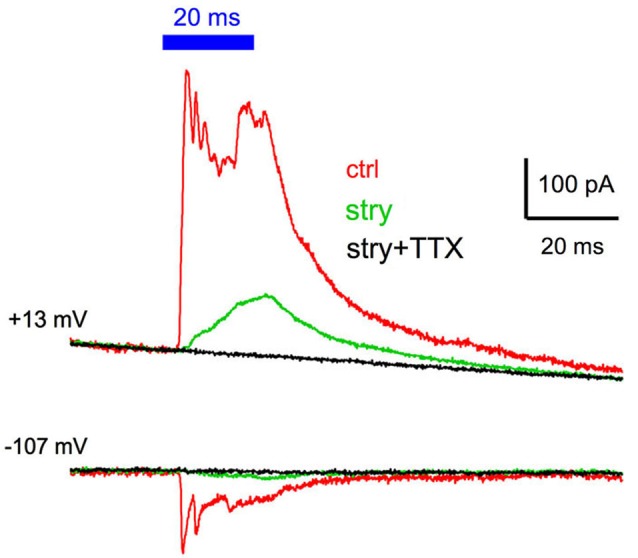
**GABAergic IPSCs are blocked by tetrodotoxin**. Cells were tested in control conditions (red traces), in the presence of 2 µM strychnine to isolate the GABAergic IPSC (green trace), and in the presence of both strychnine and 1 µM tetrodotoxin to block action potential evoked release. No IPSC was evident in the presence of tetrodotoxin in any of the three cells tested, indicating that voltage-gated sodium channel activation is required for GABA release in response to ChR2 activation.

The GABAergic IPSCs did not often show large rapidly-decaying current events typically seen with the glycinergic IPSCs, which raised a question regarding whether the IPSCs arose from action-potential evoked release, or simply from ChR2 mediated depolarization of presynaptic terminals, and subsequent asynchronous release. To address this, we tested three cells (all multipolar cells). Recordings were made in control solutions, followed by the addition of 2 µM strychnine to isolate the GABAergic component. Examples of traces for a cell held at +13 and −107 mV are shown in Figure 6. The isolated GABAergic component (green traces) was completely blocked by the addition of 1 µM TTX to the bath (black trace). The same result was obtained in the other two cells. These experiments indicate that the light evoked GABAergic IPSC requires presynaptic action potentials that initiate transmitter release, and is that ChR2-mediated depolarization of presynaptic terminals alone is not sufficient to drive release.

### Light evoked inhibiton only entrains for low frequencies

**Figure 7:**
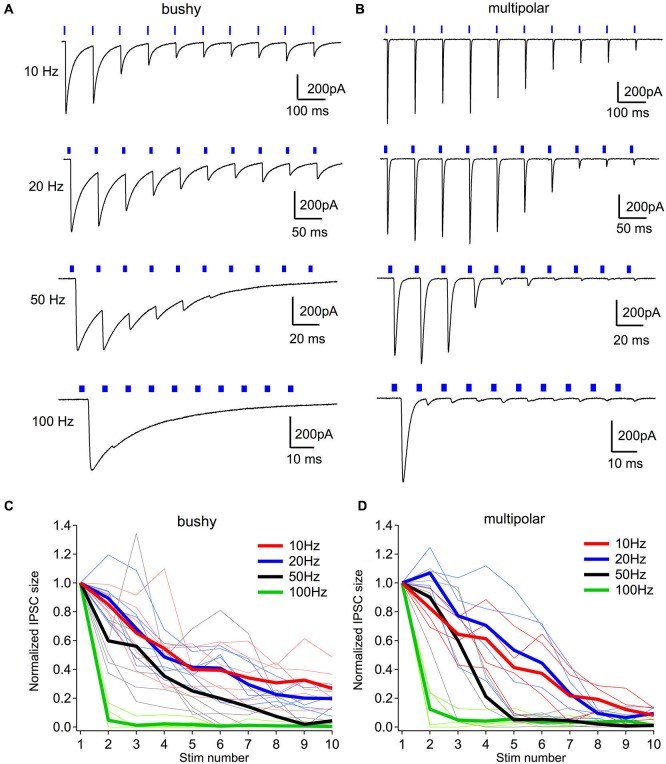
**Light evoked inhibiton only entrains for low frequencies**. **(A)** Example IPSCs to light trains consisting of ten 2-ms pulses. Notice that IPSCs are only effectively evoked throughout the trains at 20 Hz in this neuron. At 50 and 100 Hz, light failed to evoke IPSCs in the later phase of the trains. **(B)** Example IPSCs to light trains in a multipolar neuron. **(C)** Normalized IPSC amplitudes through the light stimulus trains at different frequencies in bushy neurons. **(D)** Normalized IPSC amplitudes through the light stimulus trains at different frequencies in multipolar neurons. In **(C, D)**, thin lines show individual neurons; thick lines are group averages for each frequency.

Under physiological conditions, many neurons in the the auditory system fire at relatively high rates. However, light evoked firing in inhibitory neurons in this VGAT-ChR2-EYFP mouse may not be able to follow high rates due to desensitization of ChR2 currents (Lin et al., 2009). We therefore tested the effectiveness of the light evoked inhibitory synaptic transmission with repeated stimulation. Ten light pulses of 1–2 ms duration were presented at frequencies of 10, 20, 50 and 100 Hz to drive inhbitory synaptic transmission onto bushy and multipolar neurons. Cells were held at −57 mV while using a Cs-based electrode solution with 38 mM chloride. No strychnine or GABAzine was used in this set of experiments, and excitatory transmission was blocked by including 5 µM CNQX in the bath. Under these conditions (brief light pulse stimulation), the IPSCs were only inward as the outward GABAergic IPSCs were masked by the larger glycinergic IPSCs (Figures 7A,B).

At low frequencies, 10 and 20 Hz, the brief pulses of light evoked a sustained train of IPSCs in both bushy (Figure 7A) and multipolar neurons (Figure 7B). The IPSCs became smaller during the trains in both cell types (Figures 7C,D), likely because of synaptic depression of the currents. At higher frequencies, such as 50 and 100 Hz, however, light pulses only evoked IPSCs in response to the first few stimuli, and failed to consistently drive synaptic currents later in the trains (Figures 7C,D).

## Discussion

Cochlear nucleus neurons receive both glycinergic and GABAergic inhibition from multiple sources. Glycinergic inhibition arises from local radiate multipolar (D-stellate) neurons within AVCN (Smith and Rhode, 1989; Arnott et al., 2004), tuberculoventral neurons in the DCN (Wickesberg and Oertel, 1990; Saint Marie et al., 1991; Wickesberg et al., 1991; Ostapoff et al., 1997), as well as neurons in the superior olivary complex (Ostapoff et al., 1997). GABAergic inhibition to the AVCN mostly comes from the descending projections from the superior olivary complex. Our results demonstrate that evoked glycinergic and GABAergic inhibition can be identified in bushy and multipolar neurons of the VCN using optical stimulation. The results also demonstrate that GABAergic inhibition is more prominent in multipolar cells than in bushy cells, but that the time course of the synaptic conductance is similar. This differs from the results for glycinergic inhibition, which is much faster in multipolar cells, and slower in bushy cells, both when evoked optically (this study and (Campagnola and Manis, 2014)) and electrically (Xie and Manis, 2013). Furthermore we find that the reversal potentials for glycinergic and GABAergic currents are different in both multipolar and bushy neurons.

### Method and limitations

VGAT is expressed in all cells that use glycine and GABA as a neurotransmitter (Dumoulin et al., 1999; Wang et al., 2009). We have used a pharmacological approach to characterize the synaptic conductances produced by each transmitter in the VCN. Because the VGAT-ChR2-EYFP mouse expresses ChR2 in all cells that express VGAT, all inhibitory cells should be light-sensitive. As is apparent from the spatial distribution of EYFP, which is part of the ChR2 construct in these mice, ChR2 is present not only in cell bodies, but in the dendrites, axons, and synaptic terminals of all VGAT expressing cells (Figure 1). As a result, light impinging on any part of the cell could excite it and ultimately result in transmitter release at terminals. Previous work with similar constructs shows that the threshold for excitation varies with the region of the cell that is illuminated, and that generally the soma will be the lowest-threshold region (Katzel et al., 2011). However, variation in expression, channel density, and illumination factors mean that the stimulation is likely to be relatively non-specific unless other controls are available (spatial and pharmacological). In the present experiments, illumination was limited to an area of the cochlear nuclei surrounding the recorded cell, since (for most experiments) the illumination was provided through the 40X objective. This area was approximately 780 µm in diameter, and so included a large region of the cochlear nuclei. The stimulated elements may include not only presynaptic neurons located in the slice (for example, radiate multipolar cells), but also the terminals of cells whose cell bodies are further away or even no longer present in the slice. This is an advantage in that we were able to observe synaptically-mediated GABA conductances that likely arose from pathways not included in the slice. Unfortunately, it is not clear what the source of these inputs might be. In future experiments, the use of a slice preparation in which the lateral and/or ventral nuclei of the trapezoid body is included would in principal allow a more selective activation of those inhibitory inputs, without potential co-activation of other excitatory pathways as might be engaged with electrical stimulation. Laser scanning mapping (Katzel et al., 2011; Campagnola and Manis, 2014) would also be advantageous to provide better localization of source cells in such studies.

A second limitation is that only relatively low rates of stimulation can be used with ChR2, due to its desensitization with repeated or prolonged light exposure (Lin et al., 2009). This limits the ability to reliably stimulate pathways at high rates, and partially explains why we see strong depression of the synaptic responses even at relatively low frequencies (Figure 7) compared to electrical stimulation, where responses up to 400 Hz can be studied (Xie and Manis, 2013). The use of mice expressing newer and faster ChR2 constructs (Lin, 2012) could be used to stimulate at higher rates. Nonetheless, the present study showed that this mouse is useful in utilizing photostimulation to study the neural circuitry and inhibitory synatpic transmission in local brain regions.

### Synaptically evoked GABAergic conductances

Maximal GABAergic currents and conductances were larger in multipolar neurons than in bushy cells. Unlike the time course of glycinergic inhibition (Xie and Manis, 2013), there was not a clear difference in the time course of the GABAergic synaptic currents between cell types. However, we did observe a difference in the sustained IPSCs with long-duration illumination between glycinergic and GABAergic conductances, which could reflect the relatively fast desensitization of glycine receptors (Harty and Manis, 1998) compared to the slower desensitization of GABA_A_ receptors (Frosch et al., 1992). These observations, together with the differential innervation of bushy and multipolar cells from sources within the cochlear nucleus (Campagnola and Manis, 2014), suggest that different sources and kinds of inhibition are selectively targeted to bushy and multipolar cells.

There are two additional potential mechanisms for the observed differences in the sustained IPSC responses. The first is that glycinergic and GABAergic inhibition come from different presynaptic sources. The optical stimulation could result in phasic firing in glycinergic neurons, even for sustained illumination, whereas the GABAergic source neurons could fire more tonically. In this case, the time course of the glycinergic and GABAergic IPSCs would be inherited from the presynaptic firing pattern. The available recordings from the presynaptic cells within the VCN that express ChR2 however suggests that they have sustained firing during prolonged illumination (Figure 1). However, the firing pattern could be different for terminals of descending GABAergic inputs. The second potential mechanism is that the response to thes prolonged illumination is not due to action potential evoked release in the GABAergic inhibitory neurons (or their axons), but rather results from sustained depolarization of the synaptic terminals, leading to a tonic release. However, we found that TTX blocked the GABAergic IPSCs (Figure 6), indicating that the IPSCs resulted from an action-potential dependent release of transmitter.

Although the glycinergic IPSCs reversed close to the expected Cl^−^ equilibrium potential in bushy cells, in multipolar neurons the reversal was surprisingly negative to the expected potential. Previous studies have shown that glycinergic (Wu and Oertel, 1986; Harty and Manis, 1996) and GABAergic (Wu and Oertel, 1986; Milenković et al., 2007) conductances in VCN neurons are mediated via a change in Cl^−^ conductance. There are two potential mechanisms that could contribute to a difference between the expected and measured equilibrium potentials. First, this could result from the limitations of space-clamp with single-electrode voltage clamp methods, if the inhibitory synapses are located remotely from the cell body. With a single-electrode voltage clamp, errors are introduced into the measurement of remote synaptic currents, and these errors increase with distance from the synapse to the somatic recording site (Spruston et al., 1993). Such errors can affect the estimation of reversal potentials, since the distant synaptic sites can be at a significantly different voltage than the soma. Second, recent evidence suggests that impermeant negative charges associated with intracellular phosphoproteins and surface glycoproteins can significantly affect the equilibrium for Cl^−^ (Glykys et al., 2014), and so the local ionic environment near the receptors may not be the same as that expected from the intracellular and extracellular ion concentrations.

We also unexpectedly observed a difference between the reversal potentials for glycinergic and GABAergic IPSCs. GABAergic IPSCs reversed at a potential 9 mV negative to glycinergic IPSCs in bushy cells, and 14 mV negative in multipolar cells. There are a number of potential causes for this difference. First, GABA receptors have different ionic permeability than glycine receptors for ions other than Cl^−^. In particular, of the anionic species in the ACSF and intracellularly, ${\text{HCO}}_{3}^{-}$ is more highly permeable in GABA than glycine receptors (Bormann et al., 1987). A difference in the local pH or in ${\text{HCO}}_{3}^{-}$ handling near the receptors could influence the balance of anionic species permeating the open receptor, and this could affect the reversal, as has been shown for activity-dependent shifts in cartwheel cells (Kim and Trussell, 2009). Second, the glycinergic and GABAergic synapses could have different spatial distributions, so that, again, the voltages could be different for receptors at different locations. A spatial separation between GABA and glycine receptors has been qualitatively indicated in multipolar neurons (Juiz et al., 1989, 1996). Here, glycine receptors were found mostly on the proximal dendrites, whereas GABA receptors were mostly observed in remote, medium and small caliber dendrites, some of which could belong to multipolar cells. Evidence for a similar spatial separation between GABA and glycine receptors may also hold for AVCN bushy neurons. Glycine receptors are primarily found on the soma of the bushy cells (Altschuler et al., 1986; Wenthold et al., 1988). In contrast, GABA receptors were not seen opposing axosomatic terminals in bushy cells (Juiz et al., 1989, 1996), and were reported to be present at low levels (Lim et al., 2000). Furthermore, VGAT positive puncta have been seen in alignment with bushy-cell dendrites (Gomez-Nieto and Rubio, 2009), suggesting the possibility of dendritic GABAergic synapses. Finally, as [Cl^−^]_i_ has been shown to vary in different compartments of some neurons (Duebel et al., 2006; Szabadics et al., 2006; Glykys et al., 2014), spatial segregation of GABAergic and glycinergic synapses could result in synapses faced with different ionic environments, and thus have different reversal potentials. A distal location for the GABAergic synapses would also be consistent with the slower and smaller IPSCs that we observed.

### Summary

Neurons of the cochlear nuclei receive both glycinergic and GABAergic inhibition from multiple sources. Although the synaptic properties and function of the glycinergic inhibition in the AVCN has been well studied, the synaptic function of GABAergic inhibition is less well understood because it is weaker and largely arises from sources outside the CN. In this exploratory study, we used cochlear nucleus slices from transgenic VGAT-ChR2-EYFP mouse and photostimulation to activate inhibitory neurons to study both glycinergic and GABAergic inhibition. We found that multipolar neurons receive stronger GABAergic inhibition than bushy cells, and that the time course of inhibition for both cell types was slow relative to the fast glycinergic inhibition in multipolar cells. Lastly, we observed differences in the reversal potentials for glycinergic and GABAergic IPSCs that may be consistent with different spatial distributions of receptors in the cells.

## Conflict of interest statement

The authors declare that the research was conducted in the absence of any commercial or financial relationships that could be construed as a potential conflict of interest.

## Footnotes

^1^www.numpy.org

^2^www.scipy.org
